# Efficacy and safety following two or more years of vagus nerve stimulation (VNS Therapy) in pediatric patients with drug‐resistant epilepsy enrolled in a Russian VNS Registry

**DOI:** 10.1002/brb3.3076

**Published:** 2023-05-30

**Authors:** Anna A. Feygina, Yana N. Koshelyaevskaya, Maxine Dibué, Kira V. Voronkova, Mikhail N. Klochkov, Nadezhda Y. Koroleva, Stanislav S. Ivanov, Ekaterina S. Bolshakova, Elza F. Fatykhova

**Affiliations:** ^1^ Medical Affairs International, Neuromodulation LivaNova PLC London UK; ^2^ Department of Biostatistics Regional United System of Medical Informatization, LLC Moscow Russia; ^3^ Faculty of Additional Professional Education Pirogov Russian National Research Medical University Moscow Russia; ^4^ Neurology Department Bekhterev National Medical Research Center for Psychiatry and Neurology Saint Petersburg Russia; ^5^ Head of Center for Epileptology, Neurology, and Video‐EEG monitoring, Bekhtereva Institute of the Human Brain Russian Academy of Sciences Saint Petersburg Russia; ^6^ Neurosurgery Department Republican Children's Clinical Hospital Republic of Bashkortostan Russia; ^7^ Neurology Department Voyno‐Yasenetskiy Scientific and Practical Center of Specialized Medical Care for Children Moscow Russia; ^8^ Neurosurgery Department Republican Clinical Hospital Kazan Russia

**Keywords:** drug‐resistant epilepsy, pediatric patients, Russia, seizure frequency, vagus nerve stimulation, VNS Therapy

## Abstract

**Introduction:**

Following approval in 2009 of vagus nerve stimulation (VNS Therapy) for drug‐resistant epilepsy (DRE) in the Russian Federation, this is the first multicenter study across Russia to evaluate the safety and efficacy of adjunctive VNS Therapy.

**Methods:**

The retrospective, observational registry included 58 pediatric patients with DRE (5–17 years old at implantation) who had ≥2 years of VNS. To ensure a robust evaluation process, changes in seizure frequency were evaluated for all seizure types as well as “most disabling” seizures (defined as seizures accompanied by falls, physical trauma, and/or incontinence in the absence of preventative measures).

**Results:**

With 2 years of VNS Therapy, 37 of 49 patients (76%) experiencing the most disabling epileptic seizures had a >50% decrease in frequency of such seizures, and 16 (33%) reported no longer experiencing the “most disabling” seizure type. In addition, based on the McHugh Outcome scale, VNS Therapy had a positive outcome on both frequency and severity of all epileptic seizure types, with a >50% decrease in frequency of all epileptic seizure types noted in 37 of 58 patients (64%), and 31% of patients had a Class I outcome, including 11 patients (19%) who achieved seizure freedom. VNS Therapy also had a positive effect on the frequency of status epilepticus: 13 patients (22%) had status epilepticus prior to implantation with a mean rate of 9.4 ± 17.7 events per year (range, 0–52), and after VNS Therapy, only one patient continued to experience status epilepticus (at 1 event per 4–6 months). VNS Therapy had an acceptable safety profile and no adverse events leading to VNS discontinuation were reported.

**Conclusions:**

The results demonstrate that VNS Therapy is being safely and effectively applied to pediatric patients in the Russian healthcare system.

## Graphical abstract

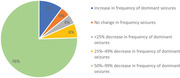

## 1 INTRODUCTION

Approximately 10.5 million children worldwide experience epileptic seizures (Guerrini, 2006; Jain & Arya, 2021; Moshe et al., 2015), and an estimated one third of the patients are resistant to antiseizure medication (ASM) resulting in high morbidity and mortality (Kwan et al., 2011). Long‐term recurrent seizures in children have a negative impact on their overall well‐being (including their physical and cognitive development), sleep cycles, mood, and quality of life and place a heavy burden on the patients, their families, and caregivers (Austin & Caplan, 2007; Guerrini, 2006; Hansen et al., 2016; Jones et al., 2010).

Vagus Nerve Stimulation Therapy (VNS Therapy^®^) involves intermittent electrical stimulation of the left cervical vagus nerve leading to stimulation of the “vagus afferent network” (Hachem et al., 2018). VNS Therapy has been approved in various countries for the treatment of patients with drug‐resistant epilepsy (DRE) with country‐specific variations in the approved indications. In the Russian Federation, VNS Therapy was approved in 2009 as an adjunctive therapy to reduce the frequency of seizures in patients experiencing epilepsy with focal seizures (with or without secondary generalization) or generalized seizures that are resistant to ASMs and who are not suitable for resective surgery.

To date, clinical studies conducted in various countries have demonstrated that following 24 months of adjunctive VNS Therapy, over 40% of pediatric patients with DRE experience ≥50% reduction in baseline seizure frequency and the response rate increases with duration of VNS Therapy (Orosz et al., 2014). Studies have also demonstrated that VNS Therapy in pediatric patients is associated with a reduction in seizure severity and seizure duration, as well as improvements in mood and certain domains of cognition (Orosz et al., 2014).

About 25 centers in Russia are currently providing VNS implantation procedures and more than 40 centers provide VNS system programing. However, the number of patients with access to VNS treatment is limited in Russia. This is due to insufficient funding of the Russian healthcare system that has led to a large gap in the provision of medical care, limited availability of modern diagnostic equipment, limited use of advanced medical technologies, an average salary for healthcare workers that is a quarter lower than the average for the national economy, and unfortunately, an overall lower life expectancy of the general Russian population compared with other industrialized nations (Ivanov & Suvorov, 2021, 2022).

To our knowledge, three articles have been published to date in the Russian language presenting findings from limited patient samples or case studies of VNS Therapy in Russian patients with DRE, and one article has been published in the English language reporting use of VNS Therapy in 17 children at a single center in Russia (Aivazyan et al., 2016; Areshkina et al., 2019; Katyshev et al., 2020; Pylaeva et al., 2019). Therefore, we initiated a multicenter registry to evaluate the safety and efficacy of adjunctive VNS Therapy in pediatric patients with DRE across the Russian Federation. We included assessment of treatment outcomes based on the most disabling seizures (defined as seizures that are accompanied by falls, physical trauma, and/or incontinence in the absence of preventative measures) as well as all types of seizures.

## 2 MATERIALS AND METHODS

### 2.1 Patient population

Patients with DRE receiving treatment with adjunctive VNS Therapy were enrolled in a multicenter, retrospective, observational registry that included eight academic and institutional research centers in the Russian Federation with dedicated epilepsy departments. Six of these centers specialized in pediatric care. The registry was approved by an institutional review board, and written informed consent was obtained from all study patients (or their legal guardians) after the procedures had been fully explained. Participating patients were implanted between January 2012 and October 2018.

Patients were recruited by physician referral from the participating centers. The registry included pediatric patients between 2 and 17 years of age at implantation; with a history of DRE characterized by epileptic seizures resistant to ASM at the time of implantation; have medical records with information on seizure types, frequency, and severity for at least 2 months prior to implantation; achieve an output current of at least 1.5 mA within 9 months of implantation; and have medical records with data from follow‐up assessments for at least the first 2 years of VNS treatment. A target of ≥1.5 mA output current was selected as it is considered to be the minimally effective current for positive outcomes (LivaNova, 2021). At an under‐therapeutic output current, the probability is lower to gain seizure freedom (Fahoum et al., 2022).

Information on follow‐up assessments included seizure types, seizure frequency, and seizure severity; adverse events and serious adverse events; use of other ASMs; changes in VNS Therapy parameter settings; and use and effectiveness of the external magnet.

Patients who underwent epilepsy surgery in the first year after VNS implantation or participated in another clinical trial within the first 2 years of implantation were ineligible to enroll in the registry.

### 2.2 Assessment of “most disabling” types of seizures and “all” types of seizures

Seizure frequency was calculated based on patient and caretaker reports. To ensure a robust evaluation process, changes in seizure frequency were evaluated for “all” seizure types as well as “most disabling” seizures.

The frequency dynamics of “all” seizure types were assessed using the McHugh Outcome scale that is a modification of the Engel Epilepsy Surgery Outcome Scale designed to assess outcomes after epilepsy surgery (McHugh et al., 2007). The McHugh is a scale of choice of VNS Therapy experts as it has been tailored to evaluate the outcomes following VNS Therapy to not only assess reduction in seizure frequency but also incorporate improvement in ictal and postictal seizure severity and benefits from use of the external magnet—both of which are very important VNS outcome measures (McHugh et al., 2007).

The “most disabling” seizures were defined as seizures accompanied by falls, physical trauma, and/or incontinence, in the absence of preventative measures.

### 2.3 Seizure severity assessment

To assess the effects on seizure severity, the validated National Hospital Seizure Severity Scale (NHS3) was adapted for this study (O'Donoghue et al., 1996). The NHS3 is widely used in Russia and there are no alternative tools available. The adapted questionnaire included items related to convulsions, falls, destructive actions, and postictal recovery period. Health professionals administered the questionnaire in the form of an interview with the patients and/or witnesses to the seizures. Data on several seizure‐related factors following implantation were collected, including changes in the severity of the most disabling seizures; changes in the most disabling (dominant) seizure type; changes in seizure duration and if there were any time correlation between change in seizure duration and length of VNS use; and changes in seizure severity and if there were any time correlation between change in seizure severity and length of VNS use.

### 2.4 Statistical analysis

Summary descriptive statistics were generated for continuous variables, and frequencies and percentages were generated for categorical variables. There was no imputation of missing data.

Pairwise comparison of means was performed using analysis of variance (ANOVA). Any significant difference was determined using Tukey's honest significant difference (HSD) test for multiple comparisons.

## 3 RESULTS

### 3.1 Demographics and baseline characteristics

The registry included 58 patients with 31 (53%) females and 27 (47%) males (Table 1). The mean age at implantation was 11 ± 3 years (range, 5–17 years) and all patients were of White race. The mean duration of epilepsy at the time of implantation was 70 ± 32 months (range, 9–166 months). Based on the current clinical guidelines in Russia, all patients were evaluated for epilepsy surgery prior to consideration of VNS Therapy.

**TABLE 1:** Baseline demographics, disease characteristics, and ASM use in the registry patients who received VNS Therapy for at least 2 years.

Characteristic	(*N* = 58)
Age at implantation	
Mean age, years (±SD)	11 (±3)
Range (minimum, maximum)	5, 17
Sex	
Female	31 (53%)
Male	27 (47%)
Race	
White	58 (100%)
Duration of epilepsy at implantation	
Mean, months (±SD)	70 (±32)
Range (minimum, maximum)	9, 166
Type of epilepsy, *n* (%)	
Focal epilepsy	29 (50%)
Combined focal and generalized epilepsy	12 (20%)
Generalized epilepsy	8 (14%)
Unknown	9 (16%)
Etiological classification, *n* (%)	
Genetic	15 (26%)
Structural–metabolic disorders	14 (24%)
Infections of the central nervous system	2 (3%)
Unknown	27 (47%)
Primary complaint	
Seizure frequency	57 (98%)
Emotional, intellectual, and speech developmental disorders	30 (57%)
Behavioral disorders	29 (50%)
Motor disorders	13 (22%)
Irritability	6 (10%)
Headache	4 (7%)
Sleep disturbance	4 (7%)
Appetite disorder	3 (5%)
Nausea	1 (2%)
Antiseizure medication (ASM) load at baseline	
4 ASMs	2 (3%)
3 ASMs	14 (24%)
2 ASMs	33 (57%)
1 ASM	8 (14%)
Missing data	1 (2%)

*Note*: Values are mean (*SD*) or *n* (%), unless otherwise noted.

All registry patients had received VNS Therapy for at least 2 years or more per the entry criteria for the registry. The analysis included follow‐up data ranging from 24 to 101 months after implantation.

As listed in Table 1, the majority of registry patients were diagnosed with focal epilepsy (*n* = 29; 50%). Other types of epilepsy were combined focal and generalized epilepsy (*n* = 12; 20%) and generalized epilepsy (*n* = 8; 14%). The epilepsy type was unknown in nine patients (16%).

Assigning causation in a heterogeneous disorder such as epilepsy can be complicated, and more than one epilepsy etiology was reported for some of the patients. Overall, the epilepsy etiologies reported by the patients included genetic disorders (*n* = 15; 26%), structural disorders (*n* = 14; 24%), and infectious causes (*n* = 2; 3%), and epileptic seizures in 27 patients (47%) were of unknown etiology.

A total of 49 patients experienced focal seizures (85%) and nine patients experienced generalized seizures (15%). The various types of seizures are listed in Table 2.

**TABLE 2:** Seizure classification in the pediatric patients in the registry.

Seizure classification	Number of patients
Focal and unknown onset seizures	49 (85%)
Automatisms, behavior arrest	2 (3.4%)
Automatisms, secondary generalized seizure	2 (3.4%)
Atonic seizure, secondary generalized seizure	1 (1.7%)
Hyperkinetic seizure	1 (1.7%)
Hyperkinetic seizure, behavior arrest	2 (3.4%)
Behavior arrest	3 (5.2%)
Behavior arrest, secondary generalized seizure	7 (12.1%)
Clonic seizure, myoclonic seizure	1 (1.7%)
Clonic seizure, tonic seizure, secondary generalized seizure	1 (1.7%)
Clonic seizure, secondary generalized seizure	1 (1.7%)
Myoclonic seizure, behavior arrest	5 (8.6%)
Myoclonic seizure, secondary generalized seizure	1 (1.7%)
Tonic seizure	4 (6.9%)
Tonic attack, behavior arrest	1 (1.7%)
Tonic seizure, behavior arrest, secondary generalized seizure	1 (1.7%)
Tonic seizure, secondary generalized seizure	2 (3.4%)
Secondary generalized seizure	11 (19.0%)
Epileptic spasms	1 (1.7%)
Epileptic spasms, tonic seizure	1 (1.7%)
Epileptic spasms, secondary generalized seizure	1 (1.7%)
Generalized seizures	9 (15%)
Generalized motor seizures	5 (8.6%)
Generalized nonmotor seizures (typical and atypical absences)	4 (6.9%)

Most patients (*n* = 57; 98%) reported seizure frequency as their primary complaint. Other complaints included emotional/intellectual/speech developmental disorders (*n* = 30; 57%), behavioral disorders (*n* = 29; 50%), motor disorders (*n* = 13; 22%), and irritability (*n* = 6; 10%). Other complaints that were reported by less than 10% of the patients were headache, sleep disturbance, appetite disorder, and nausea.

Most of the patients (98%) were receiving one to four ASMs during the time of the VNS implantation. Six of the 58 patients had also received nonpharmacological treatments prior to implantation including three patients with resective surgery, two patients had received ketogenic diet, and one patient had palliative cranial surgery (callosotomy).

### 3.2 Changes in frequency of the most disabling seizure types

The changes in the frequency and severity of the “most disabling” type of epileptic seizures (defined as seizures that were accompanied with falls, physical trauma, and/or incontinence in the absence of preventative measures) were assessed for the baseline levels (at implantation) compared with the available data from 24 months and any timepoint later than 24 months postimplantation.

**FIGURE 1:**
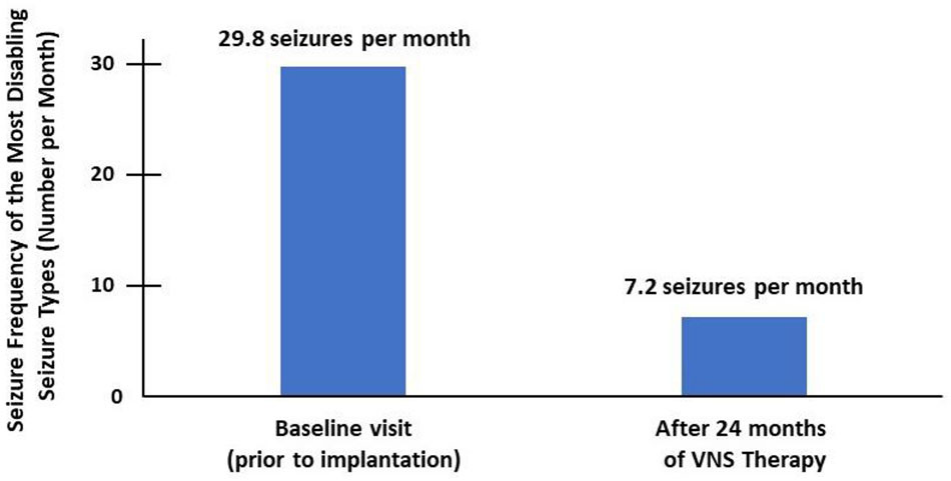
Frequency of the most disabling seizure type decreased significantly from a mean of 29.8 seizures per month at baseline to 7.2 seizures per month following 24 months of VNS Therapy (*p* < .001).

**FIGURE 2:**
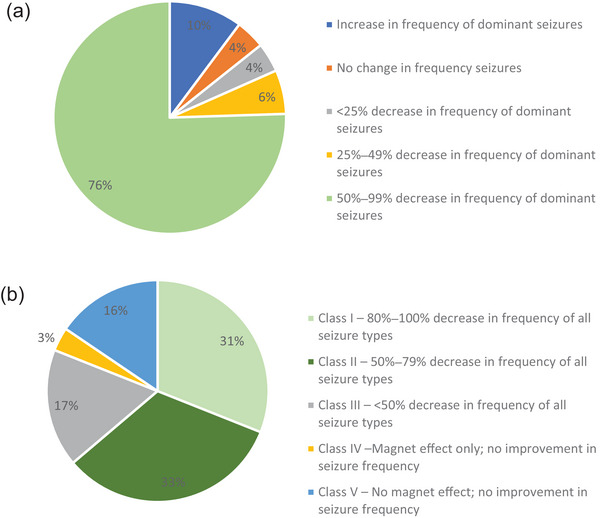
Dynamics of the most disabling seizures and all seizure types with ≥2 years of adjunctive VNS Therapy. (a) Out of 49 patients who experienced the most disabling seizures, 37 patients (76%) had 50%–99% decrease in frequency of such seizures following VNS Therapy. (b) Out of the total 58 patients, 37 patients (64%) had over 50% decrease in frequency of all seizure types following VNS Therapy (Class I and Class II outcomes according to the McHugh Outcome scale).

Of the 58 pediatric patients, 48 had a history of experiencing the most disabling seizure type and an additional patient who did not experience disabling seizures at baseline reported such seizures 2 years after starting VNS Therapy. Overall, these patients experienced a significant decrease in the frequency of the most disabling seizure type following VNS Therapy from a mean of 29.8 seizures per month at implantation to a mean of 7.2 seizures per month after 24 months of treatment (*p* < .001) (Figure 1). Notably at 24 months after implantation, 16 of the 49 patients (33%) reported that they no longer experienced the “most disabling” seizure type. In addition, 37 of 49 patients (76%) experienced a 50%−99% decrease in the frequency of the disabling epileptic seizures, three patients (6%) experienced a 25%−49% decrease, two patients (4%) experienced <25% decrease, and two patients (4%) did not experience any change in the frequency of the most disabling seizures (Figure 2a). Five patients (10%) reported an increase in the frequency of such seizures (including the patient mentioned above who did not have disabling seizures before implantation but reported them 2 years after implantation).

### 3.3 Changes in frequency and severity of all seizure types based on McHugh Outcome scale

Based on the McHugh Outcome scale, patient outcomes were classified into five levels of seizure reduction (Classes I–V). Classes I, II, and III were further subdivided into A or B according to the effects in relation to ictal and/or postictal severity.

As presented in Figure 2b, the frequency dynamics of all types of epileptic seizures with 24 months of VNS Therapy resulted in 18 patients (31%) with a Class I outcome (80%–100% reduction in seizure frequency), 19 patients (33%) with a Class II outcome (50%–79% reduction), and 10 patients (17%) with a Class III outcome (<50% reduction), and two patients (3%) had a Class IV outcome (benefited only from the external magnet). The remaining nine patients (16%) had a Class V outcome (i.e., did not experience any improvements in seizure frequency and no magnet effects) and of these, four patients (7%) reported worsening symptoms.

Importantly, 11 of the patients with a Class I outcome experienced a complete remission (i.e., were free of all types of seizures). Therefore, 19% of the analysis population achieved remission.

A total of 43 patients had information on severity of all seizure types after 24 months of implantation based on McHugh Outcome scale (Table 3). Of the other 15 patients, 11 became seizure free and four did not have information on seizure severity. (Note: The portion of Class I outcome in groups that had information on severity of all seizure types [*N* = 43] is lower than that in total group as the patients who were continuing to suffer from seizures came to additional clinic visits.)

**TABLE 3:** Changes in seizure severity of “all” seizure types with 24 months of adjunctive VNS Therapy based on the McHugh Outcome scale.

	Number of patients (*N* = 43)
Class I: 80%−100% reduction in seizure severity	7 (16.3%)
Class IA: improved ictal or postictal severity	4 (9.3%)
Class IB: no improvement in ictal or postictal severity	3 (6.9%)
Class II: 50%−79% reduction in seizure severity	18 (41.9%)
Class IIA: improved ictal or postictal severity	6 (14.0%)
Class IIB: no improvement in ictal or postictal severity	12 (27.9%)
Class III: <50% reduction in seizure severity	10 (23.3%)
Class IIIA: improved ictal or postictal severity	2 (4.7%)
Class IIIB: no improvement in ictal or postictal severity	8 (18.6%)
Class IV: Magnet benefit only	0
Class V: No improvement	8 (18.6%)

Information on the use of extra on‐demand stimulation with an external handheld magnet was available for 42 patients. The patients reported that using the magnet helped with seizure prevention (*n* = 41; 97.6%), seizure control (*n* = 41; 97.6%), reducing the duration of an attack (*n* = 38; 90.5%), and reducing the recovery time after seizures (*n* = 12; 28.6%).

### 3.4 Seizure severity

The questionnaire to assess seizure severity was completed by 43 patients who participated in an additional study visit after 24 months following implantation. A final score was generated based on the data on seizure‐related factors, and the results demonstrated that the severity of epileptic seizures and postseizure states in pediatric patients with adjunctive VNS Therapy decreased by 44%.

### 3.5 Status epilepticus

Status epilepticus is a common neurological emergency in pediatric patients. Therefore, we assessed the presence of status epilepticus defined as a seizure with 5 min or more of continuous clinical and/or electrographic seizure activity or recurrent seizure activity without recovery between seizures (Wylie et al., 2022). Status epilepticus was noted in 13 patients (22%) prior to implantation with a mean number of 9.4 ± 17.7 (range, 0–52) status epilepticus per year in these patients. After implantation, status epilepticus was reported in only one patient with an average of about 1 event per 4–6 months.

### 3.6 Usage of antiseizure medications

At the time of implantation, two (3%) patients were receiving four ASMs, 14 (24%) were receiving three ASMs, 33 (57%) were receiving two ASMs, and eight (14%) were receiving one ASM (Table 1). The most prescribed ASMs included valproic acid (*n* = 39; 67.2%), levetiracetam (*n* = 15; 25.8%), lamotrigine (*n* = 13; 22%), and topiramate (*n* = 10; 17.2%). Information on number of concomitant ASMs was not available for one patient. Mean number of ASMs before the implantation was 2.2 ± 17.7 (range, 1–4). Following 24 months of VNS Therapy, the mean number of ASMs decreased to 1.9 ± 0.9 (range, 0–4). There was no significant change in the mean number of ASMs (*p* = .064, paired *t*‐test); however, it should be noted that six patients stopped taking ASMs after treatment with VNS Therapy.

### 3.7 VNS parameters

We studied the effect of the stimulation parameters on the effectiveness of VNS Therapy, including output current (mA), signal frequency (Hz), pulse width (ms), and duty cycle (%) defined as (ON time + 2 s of ramp‐up time + 2 s of ramp‐down time) / (ON time + OFF time [s]) × 100%.

The mean, the standard deviation of the mean, and the minimum–maximum range of various parameters are presented in Table 4.

**TABLE 4:** VNS Therapy parameter settings over the course of treatment.

VNS device activation time		Output current (mА)	ON time (s)	OFF time (min)	Duty cycle (%)	Signal frequency (Hz)	Pulse width (ms)
First day VNS device was activated	Number of patients	38	38	38	38	38	38
	Mean (± SD)	0.25 ± 0.0	30 ± 0.0	5 ± 0.0	10 ± 0.0	25.9 ± 4.9	407.9 ± 122.2
	Range (min, max)	0.25, 0.25	30, 30	5, 5	10, 10	20, 30	250, 500
3 months	Number of patients	39	39	39	39	39	39
	Mean (± SD)	1.0 ± 0.3	30.8 ± 4.8	4.9 ± 0.3	10.4 ± 1.6	25.6 ± 5.0	410.3 ± 121.5
	Range (min, max)	0.5, 1.5	30, 60	3, 5	10, 18	20, 30	250, 500
6 months	Number of patients	57	57	57	57	57	57
	Mean (± SD)	1.6 ± 0.2	40.0 ± 14.3	4.7 ± 0.8	13.7 ± 4.3	27.4 ± 4.4	386.0 ± 125.6
	Range (min, max)	0.75, 2.25	30, 60	1.8, 5	10, 25	20, 30	250, 500
12 months	Number of patients	58	58	58	58	58	58
	Mean (± SD)	1.8 ± 0.3	39.5 ± 14.5	4.3 ± 1.3	16.0 ± 7.1	28.2 ± 3.8	396.6 ± 124.2
	Range (min, max)	0.75, 2.5	21, 60	0.8, 5	10, 36	20, 30	250, 500
24 months	Number of patients	58	58	58	58	58	58
	Mean (± SD)	2.0 ± 0.4	38.6 ± 13.9	3.6 ± 1.5	18.8 ± 7.9	28.8 ± 3.3	400.9 ± 123.4
	Range (min, max)	1, 3	21, 60	0.8, 5	10, 44	20, 30	250, 500
Control visit >24 months	Number of patients	58	58	58	58	58	58
	Mean (± SD)	2.1 ± 0.4	37.9 ± 14.4	3.3 ± 1.6	20.6 ± 7.8	29.2 ± 2.6	419.5 ± 117.8
	Range (min, max)	1.25, 3	21, 60	0.8, 5	10, 44	20, 30	250, 500

ANOVA was utilized to evaluate VNS Therapy parameters (output current, ON time, OFF time, duty cycle, pulse frequency, and pulse width). The analysis showed that there were significant differences only for ON time (*p* = .004) among groups with different seizure frequency outcomes compared with the control visit (i.e., a visit that took place any time following 24 months after implantation) (Table 5). Tukey's HSD test indicated significant difference in mean among the seizure freedom group and the group with 50%−99% decrease in seizure frequency (*p* = .005).

**TABLE 5:** Mean stimulation parameters in participants grouped by seizure frequency outcomes and statistical analysis using analysis of variance.

	Increase in seizure frequency	No change in seizure frequency	<25% decrease in seizure frequency	25%−49% decrease in seizure frequency	50%−99% decrease in seizure frequency	Seizure freedom	*p*‐value
Output current (mА)							
Number of patients	9	2	1	9	26	11	
Mean ± SD	2.4 ± 0.3	2.1 ± 0.2	2.0 ± 0.3	2.2 ± 0.4	2.2 ± 0.5	1.9 ± 0.3	0.159
Range (min, max)	2, 2.8	2, 2.3	1.8, 2.3	1.8, 2.5	1.5, 2.8	1.5, 2.8	
ON time (s)							
Number of patients	9	2	1	9	26	11	
Mean ± SD	37.0 ± 18.3	30.0 ± 0.0	27.0 ± 5.2	50.0 ± 17.3	45.7 ± 15.4	28.7 ± 3.3	0.004
Range (min, max)	21, 60	30, 30	21, 30	30, 60	30, 60	21, 30	
OFF time (min)							
Number of patients	9	2	1	9	26	11	
Mean ± SD	2.7 ± 2.0	2.1 ± 1.3	2.2 ± 0.7	4.3 ± 1.2	3.5 ± 1.7	2.7 ± 1.4	0.306
Range (min, max)	0.8, 5	1.1, 3	1.8, 3	3, 5	0.8, 5	1.1, 5	
Duty cycle (%)							
Number of patients	9	2	1	9	26	11	
Mean ± SD	25.3 ± 9.1	25.5 ± 13.4	20.0 ± 4.6	18.3 ± 8.5	22.3 ± 7.5	19.7 ± 7.3	0.619
Range (min, max)	16, 36	16, 35	16, 25	10, 27	16, 44	10, 35	
Pulse frequency (Hz)							
Number of patients	9	2	1	9	26	11	
Mean ± SD	30.0 ± 0.0	30.0 ± 0.0	30.0 ± 0.0	30.0 ± 0.0	28.8 ± 3.1	29.3 ± 2.7	0.876
Range (min, max)	30, 30	30, 30	30, 30	30, 30	20, 30	20, 30	
Pulse width (ms)							
Number of patients	9	2	1	9	26	11	
Mean ± SD	458.3 ± 102.1	375.0 ± 176.8	500.0 ± 0.0	333.3 ± 144.3	381.0 ± 127.9	464.3 ± 90.8	0.143
Range (min, max)	250, 500	250, 500	500, 500	250, 500	250, 500	250, 500	

### 3.8 Safety findings

Device‐ or treatment‐related adverse events included the following events in one patient (1.7%) each: hoarseness at 3 months; hoarseness (*n* = 1) and paresthesia and cough (*n* = 1) at 6 months; cough at 12 months; hoarseness and cough at 24 months; and cough was reported at a control visit more than 24 months after implantation. There were no serious adverse events or adverse reactions that led to discontinuation of VNS Therapy.

## 4 DISCUSSION

Here, we have reported the efficacy and safety results from a multicenter retrospective observational registry involving pediatric patients with DRE who were treated with VNS Therapy for at least 2 years.

Several articles have been published presenting use of VNS Therapy in Russian patients with DRE based on either limited patient samples or case studies (Aivazyan et al., 2016; Areshkina et al., 2019; Katyshev et al., 2020; Pylaeva et al., 2019). To our knowledge, our study represents the first multicenter study to evaluate the safety and efficacy of treatment with adjunctive VNS Therapy in pediatric patients with DRE across the Russian Federation.

To ensure a robust evaluation process, changes in seizure frequency following implantation were evaluated for the “most disabling” epileptic seizures (defined as seizures accompanied by falls, physical trauma, and/or incontinence in the absence of preventative measures) and changes in seizure frequency and severity were evaluated for all epileptic seizure types.

About 26% of our study patients were diagnosed with genetic epilepsies. This rate appears to be higher than that in studies of VNS in a DRE population that included children and adults (Elliott et al., 2011), but it is aligned with the findings from Symonds and colleagues who identified genetic etiologies in 31% of pediatric patients under 3 years of age (Symonds et al., 2021).

The study had limitations due to having a retrospective design and for excluding patients who underwent epilepsy surgery in the first year after VNS implantation. In addition, the portion of Class I outcome in groups that had information on severity of seizures (*N* = 43) is lower (16.3%) than that in the total group (31% of 58 patients) as most patients who experienced seizure decrease or remission on VNS Therapy chose to not have additional clinic visits and patients who continued to seek clinical support for seizure management attended additional clinic visits. Additionally, the validated English‐language NHS3 that is widely used in Russia was adapted for this study but with no formal validation. However, the overall study results showed that VNS Therapy had a positive outcome in reducing the frequency of both the most disabling epileptic seizures as well as all seizure types. VNS Therapy also had a positive effect on seizure severity and status epilepticus. Furthermore, no adverse events leading to VNS discontinuation were reported.

The analysis indicates a significant difference in ON time (*p* = .004) among groups with different treatment outcome after 24 months of VNS Therapy. This finding is aligned with other publications that have noted the positive effect of duty cycle (DeGiorgio et al., 2001; Fahoum et al., 2022). It will be of interest to further investigate this finding in future studies.

VNS Therapy is programed by clinicians using an external programing device to select the right combination of adjustable parameters (output current, signal frequency, pulse width, and signal ON and OFF times) to reach the best antiseizure effects while minimizing stimulation‐induced side effects. Therefore, one of the aims of our study was to evaluate the response rates in the Russian Federation compared with studies conducted in other countries. The estimated rate of 19% of patients who experienced seizure freedom in our study is higher than the pooled prevalence estimates of 12% identified in a meta‐analysis of 101 clinical studies evaluating VNS Therapy in pediatric patients (Jain & Arya, 2021). But the proportion of patients with ≥50% decrease in frequency of all seizure types in our study was 64%, which is higher than the pooled prevalence estimates of 56% based on other studies of pediatric patients (Jain & Arya, 2021) but in alignment with other published studies (Elliott et al., 2011; Rossignol et al., 2009). The higher positive outcome in our study may have arisen from only including patients who had achieved ≥1.5 mA output current within 9 months of implantation, and the results require further investigation.

## 5 CONCLUSION

VNS Therapy is an effective and safe adjunct treatment for DRE in male and female pediatric patients.

## CONFLICT OF INTEREST STATEMENT

A.A.F. and M.D. are employees of LivaNova and M.D. holds LivaNova stock options. Y.N.K. declares no conflicts of interest. K.V.V. has received research support from LivaNova and lecture honoraria from LivaNova, PikPharma, Alcaloid, UCB, and Eisai. N.Y.K. has received lecture honorarium and research support from LivaNova, and lecture honoraria from PikPharma, Alcaloid, and Eisai. M.N.K., S.S.I., E.S.B., and E.F.F. have received research support from LivaNova.

### PEER REVIEW

The peer review history for this article is available at https://publons.com/publon/10.1002/brb3.3076.

## Data Availability

Data available upon request due to privacy and ethical restrictions.
